# Digital child health: opportunities and obstacles. A joint statement of European Academy of Paediatrics and European Confederation of Primary Care Paediatricians

**DOI:** 10.3389/fped.2023.1264829

**Published:** 2023-12-22

**Authors:** Liesbeth Siderius, Sahan Damsiri Perera, Lars Gelander, Lina Jankauskaite, Manuel Katz, Arunas Valiulis, Adamos Hadjipanayis, Laura Reali, Zachi Grossman

**Affiliations:** ^1^ Rare Care World Foundation, Loosdrecht, Netherlands; ^2^ Youth Health Care, Almere, Netherlands; ^3^ Post Graduate Institute of Medicine, University of Colombo, Colombo, Sri Lanka; ^4^ Centre of Child Health Services, Regionhälsan, Region Västra Götaland, Borås, Sweden; ^5^ Department of Pediatrics, Medical Academy, Lithuanian University of Health Sciences, Kaunas, Lithuania; ^6^ Coordinating Center for Rare and Undiagnosed Diseases, Lithuanian University of Health Sciences Hospital Kauno Klinikos, Kaunas, Lithuania; ^7^ Patient Safety Department, Meuhedet Health Services, Tel Aviv, Israel; ^8^ Goshen Foundation, Jerusalem, Israel; ^9^ Clinic of Children’s Diseases, Institute of Clinical Medicine, Medical Faculty of Vilnius University, Vilnius, Lithuania; ^10^ European Academy of Paediatrics, Brussels, Belgium; ^11^ Medical School, European University Cyprus, Nicosia, Cyprus; ^12^ Department of Paediatrics, Larnaca General Hospital, Larnaca, Cyprus; ^13^ Primary Care Pediatrician, Italian National Health System (INHS), ASL Rm1, Rome, Italy; ^14^ Department of Pediatrics, Adelson School of Medicine, Ariel University Pediatrics, Ariel, Israel; ^15^ Department of Pediatrics, Maccabi Health Care Services Pediatrics, Tel Aviv, Israel

**Keywords:** digital health, primary child healthcare, WHO, European health data space, interoperability

## Abstract

The advancement of technology and the increasing digitisation of healthcare systems have opened new opportunities to transform the delivery of child health services. The importance of interoperable electronic health data in enhancing healthcare systems and improving child health care is evident. Interoperability ensures seamless data exchange and communication among healthcare entities, providers, institutions, household and systems. Using standardised data formats, coding systems, and terminologies is crucial in achieving interoperability and overcoming the barriers of different systems, formats, and locations. Paediatricians and other child health stakeholders can effectively address data structure, coding, and terminology inconsistencies by promoting interoperability and improving data quality and accuracy of children and youth, according to guidelines of the World Health Organisation. Thus, ensure comprehensive health assessments and screenings for children, including timely follow-up and communication of results. And implement effective vaccination schedules and strategies, ensuring timely administration of vaccines and prompt response to any concerns or adverse events. Developmental milestones can be continuously monitored. This can improve care coordination, enhance decision-making, and optimise health outcomes for children. In conclusion, using interoperable electronic child health data holds great promise in advancing international child healthcare systems and enhancing the child's care and well-being. By promoting standardised data exchange, interoperability enables timely health assessments, accurate vaccination schedules, continuous monitoring of developmental milestones, coordination of care, and collaboration among child healthcare professionals and the individual or their caregiver. Embracing interoperability is essential for creating a person-centric and data-driven healthcare ecosystem where the potential of digitalisation and innovation can be fully realized.

## Introduction

In 2022, the European Commission launched the European Health Data Space (EHDS) (1, 2) as one of the central building blocks of a strong European Health Union. The EHDS will help the EU achieve a step forward in providing healthcare to people across Europe. People can control and utilise their health data in their home country or other Member States. The EHDS offers a consistent, trustworthy, and efficient framework to use health data for research, innovation, policymaking, and regulatory activities while ensuring full compliance with the EU's high data protection standards.

Concurrently, the World Health Organization (WHO) Europe released a Pocket Book of Primary Healthcare for Children and Adolescents, which offers guidelines for health promotion, disease prevention, and management from infancy to adolescence (3). This resource is intended for utilisation by doctors, nurses, and other healthcare practitioners responsible for providing care to children and adolescents within the primary healthcare setting. The book's primary objective is to enhance the diagnosis and management of prevalent conditions in children and adolescents amenable to outpatient treatment. Additionally, it plays a crucial role in improving the utilization of laboratory and other diagnostic measures and promoting the rational use of essential drugs and equipment. This is achieved by consolidating information derived from established WHO guidelines and other evidence-based sources. The quality standards governing the provision of care to children and adolescents, which are intended to be implemented universally in primary care settings, encompass the following fundamental elements: Firstly, it is of utmost importance that every child receives evidence-based care and management of illnesses following internationally recognised guidelines, including those outlined by the WHO. Secondly, the health information system must facilitate a systematic approach to data collection, analysis, and utilization, thereby enabling early and appropriate interventions to improve the healthcare provided to each child. Thirdly, in cases where a child presents with condition(s) that surpass the capacity of the available resources for effective management, it is crucial to ensure their timely referral to appropriate care, guaranteeing a seamless continuity of treatment.

The EHDS directive of the EU and WHO Europe's Pocket Book of Primary Healthcare for Children and Adolescents, if used in synergy, could contribute to significant progress towards improving the quality of care for every child in Europe. Additionally, they could concurrently stimulate and facilitate effective communication with children and their families, ensuring meaningful participation and responsiveness to their unique needs and preferences (3).

### Health equity across health systems

Health equity is achieved when everyone can attain their full potential for health and well-being. Valuing everyone equally with a focus and ongoing societal efforts to address avoidable inequalities, and to eliminate disparities in health and health care.

WHO's standards of primary child health care accepted in a common digital European format will be readable by any child health provider within European regions and across country borders. The fact that health systems in the European Union are managed in a different way according to models of service delivery, financing, and economic policies (4, 5), should not have an influence on child health outcomes. We propose that child health indicators derived from a common digital network could provide the necessary data to compare and improve health systems.

Within each system, paediatric practice should be delivered according to the European Board of Paediatrics (EBP) standards. The EBP is the Executive of the Paediatric, formally part of the European Union of Medical Specialists (UEMS) (6). The UEMS is dedicated to advancing medical specialisation through the study, advocacy, and standardisation of the highest quality training for medical specialists (7). Its overarching mission is to promote excellence in medical practice and healthcare delivery across the European Union, emphasising the harmonisation of standards and practices for the benefit of patient care and the medical profession. The EBP sits within the European Academy of Paediatrics. The EAP is globally active through the International Paediatric Association, which advocates globally, nationally and locally for high-quality, evidence-based and child-centred paediatric care.

## Continuity of maternal and paediatric healthcare

The WHO's Sustainable Development Goals prioritise the health of women and children. To achieve these goals, healthcare policies must adopt a comprehensive approach that integrates maternal and paediatric healthcare from pre-conception, starting with addressing girls' reproductive health (8).

The WHO Pocket Book comprehensively describes the scheduled observations on growth and development from prenatal to adolescence, emphasising the need for ongoing follow-up and monitoring (3). Assessing the pattern or trajectory of growth and development over time is crucial. Similarly, other data collected during follow-up visits should be readily accessible during each healthcare encounter or treatment session. This highlights that a single healthcare visit and the observations made on that specific date are insufficient to evaluate a child's health comprehensively. For instance, complete records of children's vaccinations are essential for planning an appropriate vaccination schedule.

In order to address the broader Sustainable Development Goal (SDG) of the United Nations agenda, particular attention should be given to enhancing vital statistics, comprehending the factors influencing changes in healthcare coverage, and improving the quality of data on early childhood development and adolescent health. SDG Target 3.8 focuses on achieving Universal Health Coverage (UHC) (9), which entails ensuring that all individuals and communities have access to necessary promotive, preventive, curative, rehabilitative, and palliative health services of sufficient quality to be effective. Additionally, UHC aims to ensure that individuals are not exposed to financial hardship when accessing health services, thus promoting equitable and affordable healthcare for all. UHC is grounded in robust, people-centred Primary Health Care (PHC), explicitly targeting disadvantaged and marginalized populations.

Gathering real-world data (RWD) on health, morbidity, and mortality could provide the data elements to monitor the SDG and UHC. RWD and Real-World Evidence are already used to regulate medicines' development, authorisation, and supervision in the European Union (10). However, European data collections differ considerably, and conducting European cross-border research can be challenging due to regulatory, economic, and cultural differences (11). As a result, the accuracy and precision of results based on RWD are negatively impacted, and misleading results or false conclusions can be generated (12). Encouragingly, most European countries have adopted or planned to develop a national child and adolescent health strategy. However, variation exists in adopting key regional strategy components and data collection (13). Harmonising data collections based on evidence-based guidelines such as the WHO Pocket Book provides a platform for monitoring the vicious cycle of health care with RWD.

Enhancing a country's analytical capacity is a crucial priority in the Countdown to 2030 initiative, as it enables improved monitoring and accountability for the health of women, children, and adolescents (14). In this regard, it could be added that the European Academy of Paediatrics (EAP) and the European Confederation of Primary Care Paediatricians (ECPCP) also underlined the same problem, which exploded in Europe after the COVID-19 pandemic (15). This underscores the pressing need to address the significant challenges faced in this domain.

## Health records

Home-based records are health documents used for the history of health services received by an individual. The record is kept in the household, in either paper or electronic format, by the individual or their caregiver. These are intended to be integrated into the health information system and complement the records maintained by health facilities. Unlike facility-based records, home-based records can be used in different facilities. When data are stored as a database record, the likelihood that home-based records serve as the reliable documented source of individuals’ health data increases. Such individual health and biological data can be utilized for healthcare and other relevant purposes (16). Personal data related to maternal and child health care become readily available and accessible at home as a tool for empowering individuals and caretakers. Despite the long-standing implementation of home-based health records, there is a lack of systematic review and summary regarding their utilisation's documented benefits and potential drawbacks (17). WHO published a guideline to address this gap by reviewing the evidence of the effects of home-based records on Maternal, Newborn, and Child Health (MNCH) outcomes and health service delivery outcomes. One of the recommendations for ongoing assessment of coverage and impact is a special database or global monitoring and implementation survey. Global monitoring could capture the types of home-based records in use, the content of these, and what population coverage is achieved. Valuable cross-country data, challenges, success factors, and ways to improve implementation will serve as a learning platform. Innovative approaches to scale up community–based PHC in improving MNCH to reach the Sustainable Development Goals are needed.

A Patient Health Record (PHR) is an electronic application that enables individuals to securely access, manage, and share their health information while ensuring privacy and confidentiality (18). The primary user of a PHR is the individual, who can utilise the application to monitor various aspects of their health and well-being, including medications, physiological measurements, and assistive products. PHRs can be designed to cater to specific preventive child health programs or targeted patient groups (19). To facilitate effective data collection from PHRs, experts can create Fast Healthcare Interoperable Resources (FHIR) profiles utilising FHIR resources. These profiles assign a semantic value from standardised vocabularies to the attributes of the resources, enabling seamless bidirectional communication with Electronic Medical Records (EMRs). It is worth noting that HL7 FHIR is the only standard that inherently supports the RESTful protocol and provides application programming interfaces (APIs) for efficient resource management and exchange (20).

Providers, including paediatricians, other specialists, general practitioners, and nurse practitioners, utilise Electronic Medical Records (EMRs) to generate and manage child health data specific to their respective practices (21–23). Ideally, a child may be included in multiple EMRs from different healthcare providers while maintaining a single Patient Health Record (PHR). Using semantically interoperable health records benefits patients and providers equally (24). Children and/or their representatives gain access to accurate health data, while providers can access the child's conditions through near-verbatim data. Enhancing the quality of health and well-being data can be achieved by promoting structural and semantic interoperability in data exchange among electronic home-based records, PHRs, and external systems such as EMRs.

## Obstacles

Utilisation of diverse standards, systems, formats, and locations by manufacturers of digital health products and providers of digital health services within Member States and/or regions creates barriers and hampers interoperability. These barriers result in inconsistencies in the data structure, coding, terminology, and associated costs due to the absence of international agreements on harmonising electronic health data. Furthermore, ensuring data quality and accuracy across multiple sources presents challenges, as missing data, errors, or incomplete records can impede data harmonisation efforts. Implementing data harmonisation strategies necessitates significant resources, including financial investments, technical infrastructure, skilled personnel, and time. Insufficient resources can considerably hinder progress in this regard. Various jurisdictions may possess different laws and regulations on data governance, consent, and ownership (25).

Consequently, individuals' access to and control over their electronic health data is limited, and significant shortcomings in the interoperability of information systems within the health domain persist. Moreover, national approaches to address these issues have limited scope and fail to tackle the EU-wide problem fully. The practical implementation of effective solutions is impeded by the prevailing low interoperability within the healthcare sector, which has predominantly been addressed through non-binding regulatory instruments. Addressing this issue is crucial, as the lack of access to critical health data undermines patient safety in healthcare settings.

## European health data space

The European Health Data Space (EHDS) aims to enhance individuals’ access to and control over their electronic health data within healthcare settings, both for primary use and secondary use purposes, such as research, innovation, policymaking, patient safety, personalized medicine, official statistics, and regulatory activities (26). Additionally, the EHDS aims to promote a uniform legal framework for developing, marketing, and utilising electronic health record systems in alignment with Union values, thereby improving the functioning of the internal market (26). Considering these objectives, it is evident that the EHDS holds significant benefits for the health and well-being of children and youth.

### The primary use of data and terminologies

Digital health data encompass various types of information within the healthcare domain. By transforming medical terminology into lexicons, coding systems, and ontologies, the content becomes computationally accessible. Computational clinical content resources facilitate the systematic processing of healthcare information, leveraging its inherent meaning. By establishing a semantically integrated health system, data sharing becomes possible among different organisations and within their internal ecosystems, ensuring that the essential meaning of the data is not lost or misunderstood (27). This semantic integration enhances the interoperability and effectiveness of healthcare information exchange.

In digital global child health, various data standards are deemed highly pertinent, each carrying distinct connotations and significance.

**Figure 1:**
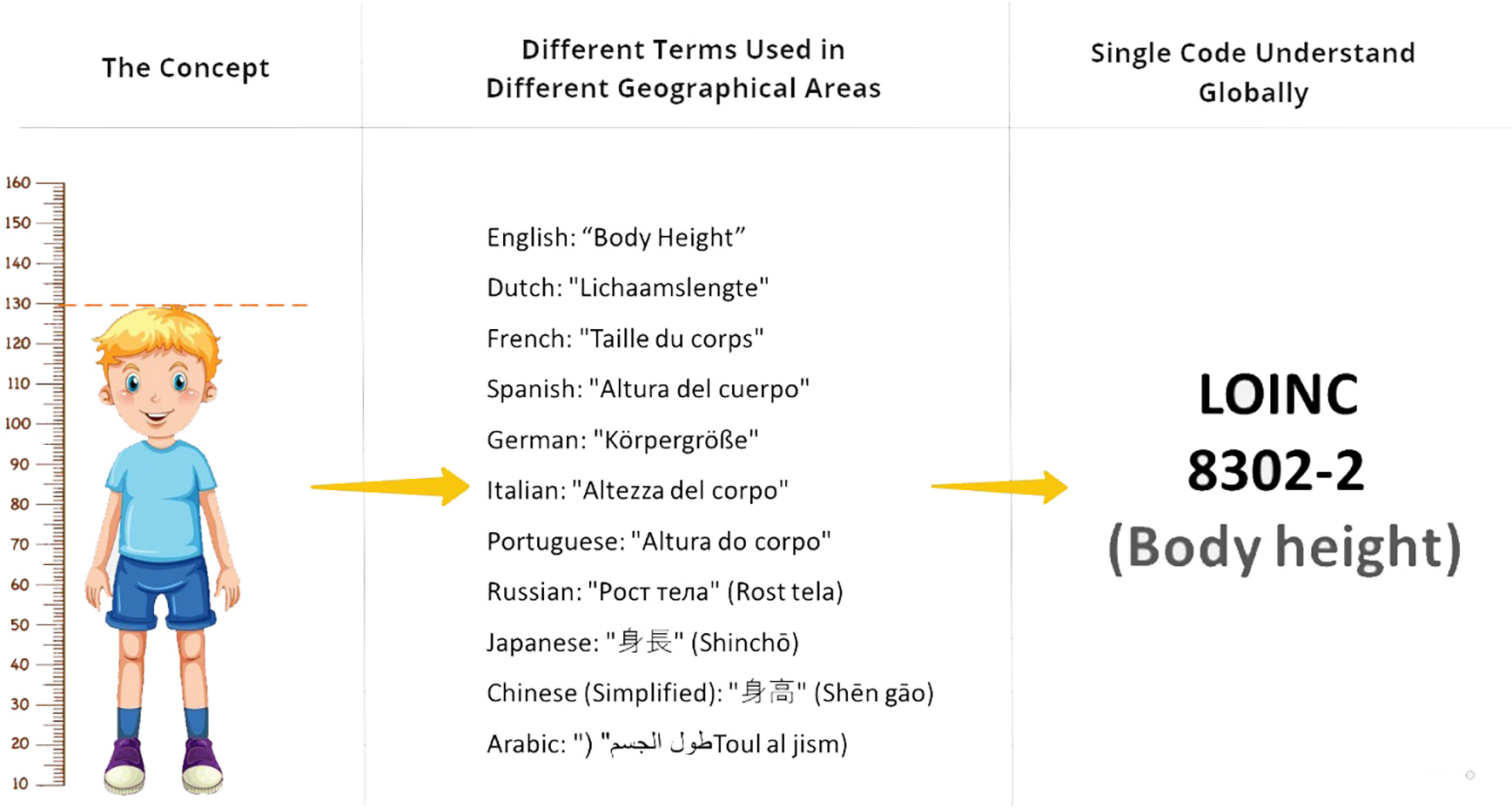
Body height coded with LOINC.

Child growth progress can be efficiently documented and shared using numerical measurements, including height, weight, and head circumference (Figure 1). This is facilitated by utilizing Logical Observation Identifiers Names and Codes (LOINC), an international standard for health measurements, observations, and documentation (28). LOINC employs a unique identifier composed of six essential elements (29). The *component* name represents the analyte or attribute being measured, such as the body weight. The *property* distinguishes different types of quantities associated with the same substance, such as mass concentration or catalytic activity. The *time* aspect identifies whether the measurement is a point in time or an interval, for example, 24H for urine sodium concentration. The *system* element specifies the specimen, body system, patient, or other objects of observation, such as cerebral spinal fluid. The *scale* element differentiates between quantitative, ordinal, nominal, or narrative text observations. Finally, the optional method element provides information on the specific approach used to generate the observation, particularly when different methods may yield clinically significant differences in interpretation.

In the global healthcare landscape, doctors measure body height in various geographical locations, using different terms such as “Lichaamslengte” in Dutch and “Körpergröße” in German (Figure 1). These variations pose challenges when sharing data internationally. Terminologies, like LOINC code “8302-2”, play a vital role in standardising the concept. Body height values are quantified numerically, accompanied by specific units of measurement, be it metric or imperial.

The availability of comprehensive measurements throughout a child's growth trajectory is crucial for detecting abnormalities. To assess the significance of a measurement, it is necessary to determine its deviation from the norm within a reference population. For instance, in primary child health, a child with achondroplasia can be identified by comparing their large head circumference and short length to those of the general population. However, during the follow-up of that child, these measurements need to be compared to those of other children with achondroplasia to identify hydrocephalus and evaluate the effectiveness of new medications. This highlights the importance of linking measurements (LOINC) (29) with diseases coded by International Classification of Disease (ICD) 10–11, enabling more accurate assessment and monitoring of the child's health status globally.

At a societal level, the data collected on weight and length can serve as valuable indicators for monitoring obesity rates and identifying cases of failure to thrive. Additionally, these measurements can be utilised to update and refine references regarding secular trends in growth patterns (secondary use of data).

In radiology, RSNA RadLex ontology is a comprehensive resource for imaging-related terms. In collaboration with the Regenstrief Institute, RSNA developed the LOINC-RSNA Radiology Playbook, establishing standardised naming and coding for imaging (30, 31). For example, a shorter fetal femur length may signal skeletal dysplasia in fetal skeletal assessment. To record fetal femur length via ultrasound, use LOINC term 11963-6, denoting “Fetal Femur diaphysis [Length] US” (32). This standardisation promotes consistent communication of imaging findings, aiding efficient analysis and interpretation in clinical practice and research.

**Figure 2:**
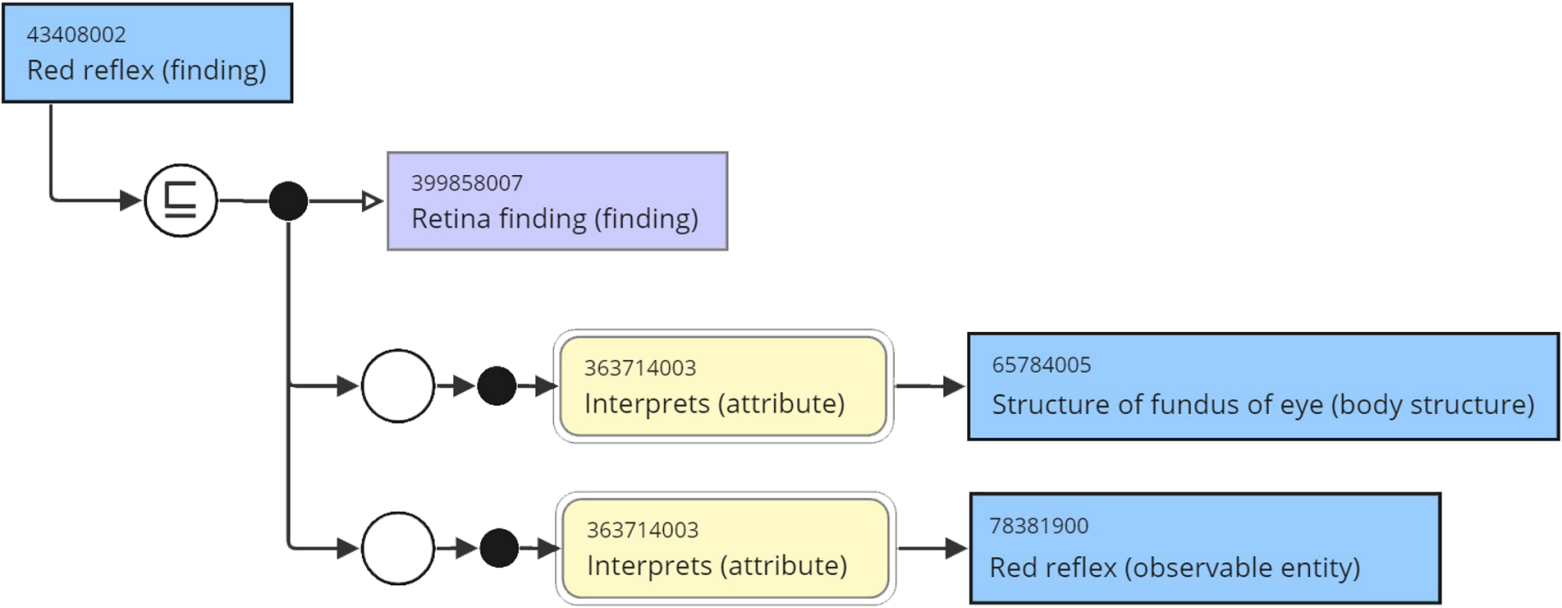
Compositional grammar: illustrating the representation of Red reflex.

Systematised Nomenclature of Medicine Clinical Terms (SNOMED CT) offers many appropriate terms. SNOMED CT concepts are described and defined by expressions following a formalism called Compositional Grammar, which can be interpreted according to description logic, allowing SNOMED CT to be considered a formal ontology (33). As an illustration, within SNOMED CT, the observation of “Red reflex” in a neonate is assigned the code 43408002 (Figure 2). However, its definition can be expanded using a compositional grammar expression. In this case, the expression identifies “Red reflex” as a finding related to the retina (code: 399858007) in the structure of the fundus of the eye (code: 65784005) and is interpreted as a red reflex (code: 783819001) by an entity (code: 363714003) (34). This detailed compositional grammar expression provides a more comprehensive understanding of the “Red reflex” within SNOMED CT, facilitating precise documentation and communication of clinical findings.

The ICD plays a vital role worldwide, providing crucial information on the global occurrence, causes, and impact of human diseases and mortality. Using ICD codes, data is reported and categorised, serving as the primary foundation for health records and disease statistics across various levels of care (35). These data and statistics are essential for healthcare payment systems, service planning, quality and safety administration, and health services research. The standardised diagnostic guidance associated with ICD categories facilitates large-scale data collection and enables extensive research. Initially developed in the 19th century, the latest version, ICD-11, was officially adopted by the 72nd World Health Assembly in 2019 and has been in effect since 1st January 2022 (35).

As the need for semantic interoperability between clinical terminologies like SNOMED CT and statistically aggregated classifications such as ICD-11 continues to grow, leveraging the SNOMED CT concept model becomes increasingly important. It offers a logical foundation for automating coordination and ensuring consistent data representation. Challenges encountered when aligning SNOMED CT with ICD highlight the importance of addressing quality issues in applying the SNOMED CT concept model (36).

The International Classification of Functioning, Disability, and Health (ICF) is a recognised standard for linking diseases or health conditions to various aspects of an individual's functioning, participation in activities (such as education and work), and the influence of environmental factors (37). It provides a comprehensive framework that complements the ICD by capturing a broader understanding of health and its impact on individuals’ lives. By considering the interplay between health conditions, functional limitations, and contextual factors, the ICF enhances health outcomes' assessment, management, and measurement, facilitating a more holistic approach to healthcare. This aligns with the historic resolution on strengthening rehabilitation in health systems, passed by the World Health Assembly in 2023 (38). The resolution emphasises enhancing health information systems by capturing rehabilitation-relevant information, including system-level rehabilitation data and functioning-related information. The ICF is recommended to ensure a comprehensive understanding of functioning and disability while emphasising data disaggregation by sex, age, disability, and other context-relevant factors (38). Compliance with data protection legislation is also crucial to safeguard privacy and enable robust monitoring of rehabilitation outcomes and coverage.

The Human Phenotype Ontology (HPO) serves as a valuable resource for representing the outcomes of observations related to abnormal phenotypes that are medically relevant (39). It provides a logically defined vocabulary that describes and classifies such phenotypes. The HPO has gained widespread acceptance as the standard for computational analysis of phenotypes in genomics and rare diseases, facilitating the identification and characterisation of genetic variations and their associated clinical manifestations.

The Human Genome Organization (HUGO) Gene Nomenclature Committee (HGNC) is the authoritative body in naming human genes (40). LOINC, a widely used health measurement and observation coding system, incorporates HGNC's terminology for naming genes. Additionally, LOINC employs the syntax the Human Genome Variation Society (HGVS) defines to encode sequence variants of interest (41). This harmonisation of terminologies and coding systems ensures accurate representation and interoperability of genetic information, supporting research, clinical practice, and the advancement of genomic medicine.

The Orpha nomenclature and Orpha code constitute a distinctive coding system designed to identify rare diseases characterised by their low prevalence (42, 43). Rare diseases number over 8,000, often with a genetic basis and an onset during childhood (44). While each disease is individually rare, collectively, they present a significant health challenge (45). The International Classification of Diseases (ICD) lacks a comprehensive code tailored for rare diseases (46). However, the Orpha nomenclature and the Orpha code prove highly valuable in addressing this gap. The Orpha code is linked to an extensive and comprehensive database, and it offers mappings to the ICD-11, further enhancing its utility in rare diseases (42, 46).

Over 85% of all European children are vaccinated and monitored by the WHO. The WHO classification system Anatomical Therapeutic Chemical (ATC) registers vaccines and medicines (47). A more detailed International Organization for Standardization (ISO) suite of IDMP (Identification of Medicinal Products) standards is coming (48). These standards provide an international framework to uniquely identify and describe medicinal products with consistent documentation and terminologies and to ensure the exchange of product information. Vaccination programs within the European region differ, and optimal coverage with recommended vaccines can be improved. Registration of vaccinations in a personal child health record allows personal data travel with the child. Moreover, accepting a global standard and uniquely identifying specific medicinal products, including vaccines, will improve safety.

**Figure 3:**
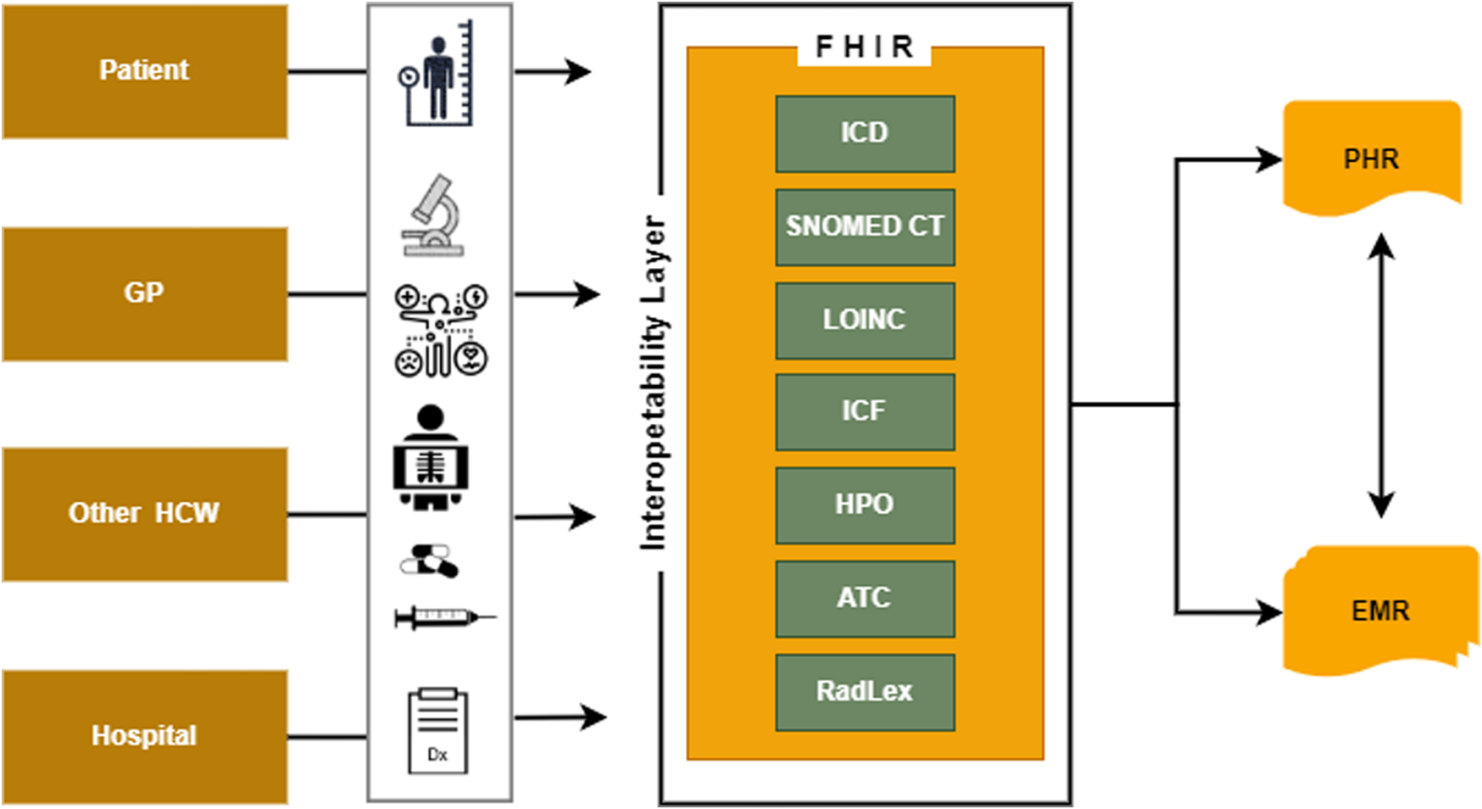
The interoperability layer necessary to exchange health information data using PHR and EMR.

The EHDS plays a pivotal role in promoting efficient exchange and access to various electronic health data types, such as electronic health records, genomics data, patient registries, and data related to social participation (Figure 3). Establishing an inclusive international framework for sharing health-specific data is crucial for advancing universal integrated child healthcare, with a particular focus on children with disabilities. Universal data collection principles significantly enhance medical care and facilitate the full participation of children with chronic and disabling conditions in society. By embracing digitalisation in healthcare, unnecessary tests and the potential harm can be minimised.

Through the implementation of this framework, significant progress can be made towards achieving universal integrated child healthcare, ensuring equitable access to quality care, and promoting the well-being of all children, regardless of their health conditions or disabilities.

Thus, medical and social guidelines for primary preventive care for all children living in Europe, including those with special needs, can be transformed into structured digital standards. To complement home-based maternal, newborn, and child health records, these sets should be developed in a multisectoral approach with clinicians and other professionals, including patient and parent organisations (14).

### Secondary use of data

The advancement of digitalisation and innovation in real-world data has created novel opportunities for developing and utilising vaccines and medicines (49). As innovative vaccines and medicine do not reach all patients across Europe at the same speed and patients might not have access to the therapeutics, they need due to shortages, digitalisation may help to control even the distribution and availability of necessary vaccines and medicines. The weaknesses exposed by the SARS-CoV-2 pandemic ask for appropriate actions to strengthen the system. Innovative technologies will reach patients in order to inform them and reduce health risks while addressing market failures.

The EHDS will help to improve understanding, prevention, early detection, diagnosis, treatment, and monitoring and help to prevent, detect, and rapidly respond to health emergencies at the global level.

### Ethical considerations

Patients possess an ethical entitlement to receive consistently high-quality care across their entire healthcare journey (50). It is imperative to establish specific guidelines for the two primary categories of health data usage: primary data usage (on healthcare delivery) and secondary data usage (for research, policy development, and private health service advancement) (50). Protecting the ethical rights of patients extends beyond mere data transfer within digital networks. There remains a potential for harm or injustice, even in cases where patients themselves are not being transferred, such as when patient health data is inappropriately shared, leading to breaches of privacy. In the context of digital health networks, this underscores the responsibility to design the network in a manner responsive to patients' medical requirements and autonomous choices (50).

Considering the high-level FAIR Guiding Principles is paramount when dealing with the secondary use of data (51). These principles provide a crucial framework for data publishers and stewards, helping them assess whether their chosen implementation methods align with the goals of making their digital research materials Findable, Accessible, Interoperable, and Reusable (FAIR). It is important to note that these FAIR Guiding Principles are overarching guidelines that come into play before specific implementation decisions are made. They do not endorse any specific technology, standard, or implementation solution or are not a standard or specification.

One distinguishing feature of the FAIR Guiding Principles is their emphasis on applying FAIRness to human- and machine-driven activities (51). This emphasis sets them apart from many other initiatives in the field and underscores their comprehensive approach to data management and accessibility.

### Cross-border exchange

Cross-border exchange of electronic health data is still minimal. That is partly explained by the significant diversity in standards applied to electronic health data in different Member States. CEN/TC 251 Health Informatics delivers and maintains health informatics standards for Europe. These include requirements on health information structure to support clinical and administrative procedures, technical methods to support interoperable systems, and safety, security, and quality requirements, such as the Technical Specification for the implementation guideline for European Use of the International Patient Summary IPS CEN (52). The European achievements align with the work of the Global Digital Health Partnership (GDHP) on an International Patient Summary (IPS) to advance efforts to have patients' health data flow across country borders (53). The urgent need for the IPS is demonstrated now more than ever. Amidst the distressing scenes of children fleeing war zones and conflict-ridden areas, we witness the destruction of hospitals and health facilities caused by military actions. Additionally, the escalating incidence of floods, fires, and hurricanes due to climate change poses a significant risk to institutional health data, potentially leading to its loss or damage.

## The way forward

The European Academy of Paediatrics promotes the right to enjoy the highest attainable standard of health and well-being for all children in Europe and in other Countries. This includes a solid commitment to ensure the most vulnerable are not left behind, including children on the run for violent conflicts and children with a chronic disabling condition. The EAP highlights the need for the involvement of child health professionals in the digitalisation of child health data. To achieve a common understanding of how the child's medical history, diagnoses, and treatment, medications, allergies, immunizations, as well as radiology images and laboratory results, should spread between different entities from the health system (general practitioners, hospitals, pharmacies, care services) becoming part of a common infrastructure to ensure connectivity and interoperability efficiently and securely. While patient portals, mobile applications, and other personal health data access services exist in many places, access to health data is limited and time-consuming, and privacy is not guaranteed. Data fragmentation negatively impacts individuals who need such access immediately due to urgent circumstances about their health condition. Hence, it is imperative to compel software providers to adhere to interoperable standards. The absence of seamless health data exchange can result in avoidable delays that can have fatal consequences.

### Opportunities

- EHDS directive of the EU and WHO Europe's Pocket Book of Primary Healthcare for Children and Adolescents could, if used in synergy, contribute to significant progress toward improving the quality of care for every child in Europe.
- Quality standards governing the provision of care to children and adolescents, which are intended to be implemented universally in primary care settings can be delivered in a uniform digital framework
- Paediatric practice in Europe is harmonised according to the European Paediatrics training standards
- Enhancing the quality of health and well-being data can be achieved by promoting structural and semantic interoperability in data exchange among electronic home-based records, PHRs, and external systems such as EMRs.
- Gathering real-world data (RWD) on health, morbidity, and mortality could provide the data elements to monitor the SDG and UHC.

### Obstacles

- Variation exists in adopting key regional strategy components and data collections.
- There is a potential for harm or injustice, when patient health data is inappropriately shared, leading to breaches of privacy.
- Medical doctors trained in informatics are sparce.
- Literacy and trust in digitalized health systems of the general public is not aphoristic.
- It is imperative to compel software providers to adhere to interoperable standards.

## Recommendations from EAP and ECPCP

The EAP and the ECPCP strongly support the development of European health dataspace and emphasise that health data regarding children and adolescents must be possible to use at every contact with healthcare wherever this contact takes place in Europe. Standardizing digital data using appropriate protocols of interoperability would make it possible to interpret the information in all computerised systems despite the different languages in Europe.

We call for creating a new generation of paediatricians and family doctors caring for children and families. Lifelong and quality health needs to be focussed on a better understanding of neuroscience, molecular biology, genomics, epidemiology, health economics, sociology, developmental psychology, and the digitalisation of health care.

## Recommendations for the authorities

○ European countries should invest in developing digital health literacy and infrastructure to support the implementation of the health data space.
○ The authorities should guarantee privacy and security as health data contain sensitive and personal information. Authorities must implement specific regulations ensuring data protection and safe data sharing.
○ In case of a request by any official organisation to access the data, this organization should provide detailed information about the use of health data. The information should include the following:
  ○ The person who is going to be authorised to access data.
  ○ The purpose of accessing the data
○ The organization that will access the data should report the results or findings of projects for which data were used in profane language in a specific period.

## Recommendations for educational services for children and adolescents

○ Provide appropriate accommodation and support services for individuals with special educational needs and disabilities.
○ Ensure comprehensive documentation of allergies, medications, and other health-related needs to facilitate efficient healthcare delivery.
○ Implement timely health screenings and assessments to identify and address potential health issues promptly.
○ Establish systems for aggregating data to support health education and promotion initiatives.
○ Enable prompt response to epidemiological hazards by establishing timely monitoring and intervention mechanisms.

## Recommendations related to parents/caregivers' rights

○ Ensure that parents have the right to access their child's health records, including comprehensive medical history, test results, and treatment information.
○ Parents can opt-out if they do not wish their child's health data to be processed for secondary use, respecting their preferences and concerns.
○ Emphasise the importance of parents taking responsibility for safeguarding the privacy and confidentiality of their child's health data, fostering a culture of trust and security in healthcare settings.

## Recommendations to paediatricians

○ Ensure comprehensive health assessments and screenings for children, including timely follow-up and communication of results. This includes early newborn screenings to detect and address potential health issues.
○ Implement effective vaccination schedules and strategies, ensuring timely administration of vaccines and prompt response to any concerns or adverse events.
○ Monitor and assess developmental milestones, continuously monitoring behavioural and mental health. Pay particular attention to signs of neglect and abuse, taking appropriate actions to protect the child's well-being.
○ Foster care coordination by collaborating with other healthcare professionals involved in the child's care. This includes sharing relevant health data with specialists, hospitals, and other healthcare facilities to ensure continuity of care and promote comprehensive treatment.
○ Prioritise integrating health information systems to enable seamless sharing of patient data and facilitate efficient collaboration among healthcare providers involved in the child's care. This can improve care coordination, enhance decision-making, and optimise health outcomes for children.

## Conclusion

In conclusion, prioritising the rights of children, their parents, and caregivers and implementing key recommendations for paediatricians is crucial for ensuring optimal healthcare for children. By granting parents access to their child's health records and allowing them to opt out of data processing, we respect their autonomy and empower them to make informed decisions. It is also essential to emphasise the responsibility of parents in safeguarding the privacy and confidentiality of their child's health data.

Paediatricians are vital in conducting comprehensive health assessments, screenings, and monitoring developmental milestones. By following timely vaccination schedules, promptly addressing concerns, and actively monitoring behavioural and mental health, paediatricians can contribute to the early detection and intervention of potential health issues. Additionally, effective coordination of care and collaboration with other healthcare professionals enable comprehensive treatment plans and seamless sharing of information, ultimately improving the overall health outcomes for children.

By embracing these recommendations and fostering a person-cantered approach, paediatricians can promote the well-being of children, ensuring they receive the necessary care, support, and interventions. Investing in integrating health information systems and promoting data sharing among healthcare providers will further enhance the quality and continuity of care. Ultimately, by prioritising the needs of children, we contribute to building a healthier future for the next generation.
